# Anesthesia management of a premature neonate during minimally invasive sclerotherapy of a large chest wall mass: A case report

**DOI:** 10.1097/MD.0000000000021726

**Published:** 2020-08-21

**Authors:** Xi Luo, Min Xie, Yushan Ma, Xiaoqin Jiang

**Affiliations:** a Department of Nursing, Sichuan Academy of medical Sciences and Sichuan Provincial People's Hospital; b Department of Operating Room; c Department of Anesthesiology, West China Second University Hospital, Sichuan University; d Key Laboratory of Birth Defects and Related Diseases of Women and Children (Sichuan University), Ministry of Education, Chengdu, Sichuan Province, China.

**Keywords:** minimally invasive sclerotherapy, perioperative respiratory adverse events, premature newborn

## Abstract

**Rationale:**

The most common critical incidents in pediatric anesthesia are perioperative respiratory adverse events (PRAE), which occur more often in neonates and account for one-third of anaesthesia-related cardiac arrests. It is crucial to maintain an open stable airway during anesthesia in neonates, as this population has a low oxygen reserve, small airways, and the loss of protective airway reflexes under general anesthesia.

**Patient concerns:**

A 6-day-old premature newborn underwent minimally invasive sclerotherapy under general anesthesia. For high-risk premature neonates, the selections of the anesthesia and airway device are extremely important, as those factors directly affect the prognosis.

**Diagnoses:**

B ultrasound and computed tomography (CT) revealed a large mass from the left chest wall to axilla, which was suspected to be a lymphocele.

**Interventions:**

Minimally invasive sclerotherapy was performed under inhalation anesthesia. After the initiation of anesthesia, a laryngeal mask was placed to control airway. Anesthesia was maintained intraoperatively via sevoflurane inhalation with spontaneous breathing. No accidental displacements or PRAE occurred.

**Outcome:**

The operation and anesthesia process was stable and safe. The patient discharged at 2 days postoperatively.

**Lessons:**

Minimally invasive sclerotherapy in a premature neonate is an operation with an extremely short operation time and minimal trauma, but a very high anesthesia risk and risk of PRAE. Anesthesia management is very important in a premature neonate undergoing a very short surgery under general anesthesia. Total sevoflurane inhalation general anesthesia and laryngeal mask airway control with spontaneous breathing may be an ideal option to reduce PRAE during very short surgery in a premature neonate.

## 1 Introduction

In paediatric anaesthesia, perioperative respiratory adverse events (PRAE) are the most common critical incidents encountered^[1–4]^ and account for one-third of anaesthesia-related cardiac arrests.^[5]^ Although the incidence of PRAE is 15% in the general paediatric population, the incidence of PRAE is doubled in infants ≤1 year old.^[6]^ It is especially crucial to maintain an open and stable airway in this infant population, due to the presence of small airways, a low oxygen reserve, and the loss of protective airway reflexes under general anesthesia.

## 2 Case report

**Figure 1:**
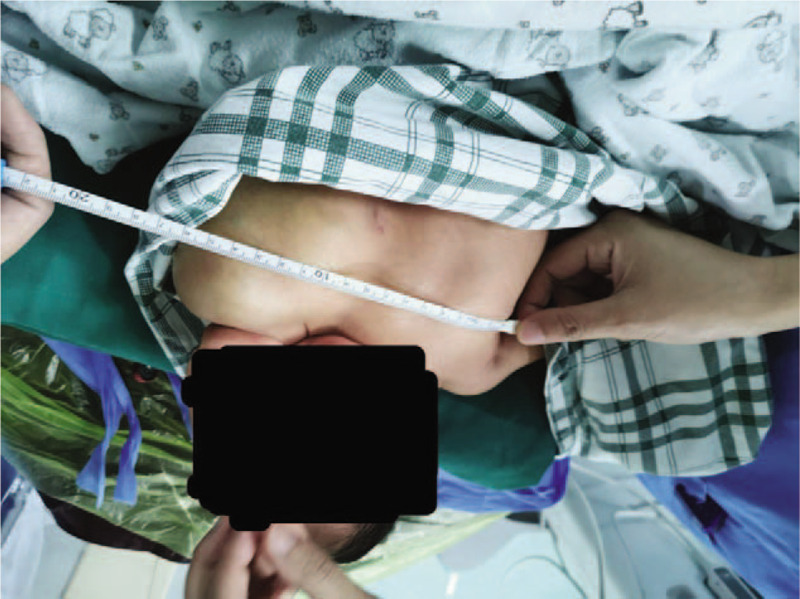
A large soft and fluctuant mass (15∗10∗10 cm) without crevasse was found on the left breast.

**Figure 2:**
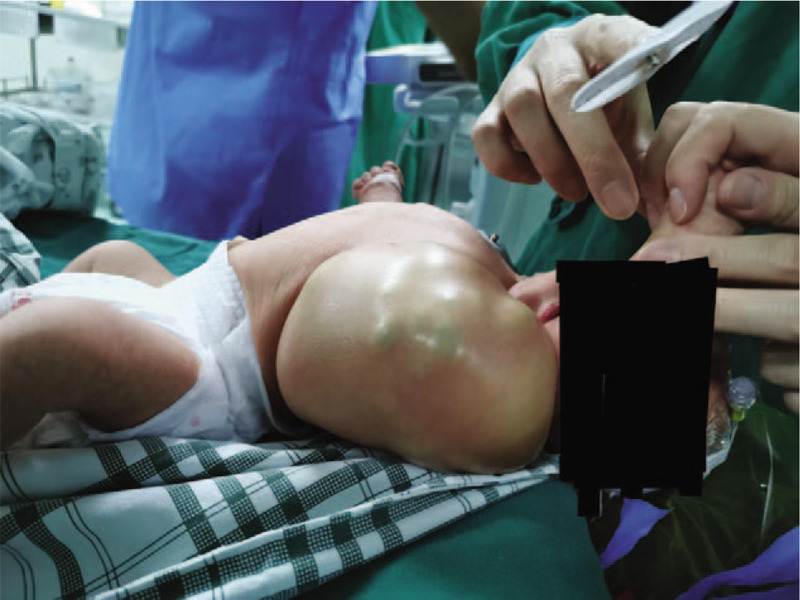
Viewed from the left side, the premature newborn has a large soft and fluctuant mass (15∗10∗10 cm) without crevasse on his left chest.

A 6-day-old premature neonate was born on May 3, 2019, by caesarean section at a gestational age of 36 + 5 weeks. The birth weight was 3270 g, and apgar score was 9-10-10. A large soft and fluctuant mass (15∗10∗10 cm) without fissures was detected by neonatologists and obstetricians after birth [Figs. 1 and 2]. The neonate was subsequently admitted to neonatology department for further therapy. CT showed that the left axilla and lateral chest wall were mainly occupied by cystic components. The large left chest wall mass was suspected to be a lymphocele. After consultation between the departments of neonatology, pediatrics and anesthesiology, the intralesional lymphangioma injection was planned under general anesthesia.

The premature neonate was routinely fasted. No preoperative medication was used. After entering ICU, the newborn was monitored by electrocardiogram, and had a heart rate 160 times/min, blood pressure 89/40 mm Hg, and SPO_2_ 99% under total inhalational general anesthesia. A neonatal breathing pipeline was selected. The anesthesia machine was set to manual mode, and the adjustable pressure-limiting valve was opened. The concentration of sevoflurane was adjusted to 8%, oxygen concentration was 100%, and fresh air flow at 6L/L was used to prefill circuit. A mask suitable for premature newborn was placed on the face for the induction of anesthesia via sevoflurane inhalation. A no. 1 laryngeal mask was placed after the eyelash reflex had disappeared. After checking that of the laryngeal mask was at the appropriate depth, the anesthesia machine was connected. The concentration of sevoflurane was adjusted to 2% to 4%, and further adjusted according to the vital signs and anesthesia depth of the newborn. Spontaneous breathing was retained, and manual assisted ventilation was provided when necessary. The concentration of terminal respiratory carbon dioxide was maintained at 35 to 45 mm Hg intraoperatively. Intralesional injection was performed under the guidance of digital subtraction angiography (DSA). Approximately 130 mL of pale yellow transparent liquid was successfully aspirated from the lesion. Under DSA monitoring, 1.5 mL of mixture of bleomycin and onecek and 40 mg of cinnamol were injected percutaneously as sclerotherapy. The operation time was 16 min. When the operation was finished, the sevoflurane inhalation was stopped, and fresh air flow was adjusted to 6 L/min. When the neonate opened his eyes, laryngeal mask was removed. The vital signs remained stable throughout the operation.

## 3 Discussion

Lymphocele may occur in premature newborns. We reported a rare case of a preterm neonate with large chest wall mass who successfully underwent minimally invasive sclerotherapy under general anesthesia. The large chest wall mass was postoperatively diagnosed as a lymphocele. Minimally invasive sclerotherapy is a new treatment for lymphocele in a neonate. This technology achieves its therapeutic purpose by ultrasound- or DSA-guided injection of a sclerosing agent (lauromacrogol) into the cyst cavity, which makes the cyst gradually shrink and finally disappear.

Minimally invasive sclerotherapy comprises minimal stimulation and an extremely short operation time, sometimes only a few minutes. These characteristics of sclerotherapy enable this procedure to be performed in premature neonates under general anesthesia, despite this population having an extremely high anesthesia risk. Premature neonates have poor tolerance to anesthesia and surgery due to the incomplete development of various systems and organs, and the recent change from the intra- to the extra-uterine environment. Neonates and premature infants are more likely to have perioperative complications than adults, and the mortality rate of pediatric anesthesia complications is 10 times higher than that of adults. The reported incidence of anesthesia-related cardiac arrest in infants is 19 to 24/10,000, which is several times higher than the incidence in all anesthetized children (1–7/10,000). The mortality rate of neonates who experience anesthetized cardiac arrest is 72%. However, a large randomized controlled trial showed that the same 5-year outcomes were achieved by infants who received general anesthesia versus conscious sedation. Furthermore, general anesthesia in the neonatal period does not damage long-term neurological development.^[7]^ General anesthesia in neonatal period does not damage long-term neurological development. Anesthesiologists must be familiar with all neonatal anesthesia techniques and choose the most appropriate anesthetics to ensure a good anesthetic effect and reduce the incidence of complications.

Sevoflurane is one of the most commonly used inhaled anesthetics in clinic.^[8]^ Its unique pharmacodynamic and pharmacokinetic characteristics make it very suitable for neonatal general anesthesia, as it is a short-acting inhalational anesthetic that produces minimal respiratory tract stimulation and achieves stable and rapid anesthesia induction, maintenance, and revival, with few postoperative complications.^[9]^ Furthermore, inhalational anesthesia enables precise breathing control or retained spontaneous respiration, with no need for muscle relaxants, while the insertion of a laryngeal mask enables effective control of the airway.^[10]^ These factors enhance the safety of general anesthesia in premature infants with risk factors such as low birth weight and low blood volume, as even mild intraoperative bleeding and/or lack of oxygen can be fatal to neonates. Thus, sevoflurane inhalational anesthesia via a laryngeal mask is recommended for short surgeries in premature neonates.

In the present case, the premature neonate did not develop into severe respiratory depression during anesthesia. However, clinicians should be aware that once serious inhibition of circulation and respiration appears during anesthesia induction in premature neonate, the inhaled anesthetic concentration should be immediately reduced, or completely stopped. The loop should then be flushed with 100% high-flow oxygen to prevent premature newborn from occurrence of serious and even potentially fetal complications.

## 4 Conclusions

Minimally invasive sclerotherapy in a premature neonate is an operation with an extremely short operation time and minimal trauma, but a very high anesthesia risk and risk of PRAE. Total sevoflurane inhalation general anesthesia and laryngeal mask airway control with spontaneous breathing may be an ideal option to reduce PRAE in a premature neonate.

## Author contributions

**Data collection:** Min Xie, Yushan Ma.

**Writing – original draft:** Yushan Ma.

**Writing – review & editing:** Xi Luo, Xiaoqin Jiang.

All authors have read and approved the final manuscript.

## Correction

When originally published, the affiliations appeared out of order as “a Department of anesthesiology, West China Second University Hospital, Sichuan University, Chengdu, Sichuan Province, b Department of Nursing, Sichuan Academy of medical Sciences and Sichuan Provincial People's Hospital, c Key Laboratory of Birth Defects and Related Diseases of Women and Children (Sichuan University), Ministry of Education, d Department of Operating Room, West China Second University Hospital, Sichuan University, Chengdu, Sichuan Province, China." These have since been corrected to ” a Department of Nursing, Sichuan Academy of medical Sciences and Sichuan Provincial People's Hospital, b Department of Operating Room, c Department of Anesthesiology, West China Second University Hospital, Sichuan University, d Key Laboratory of Birth Defects and Related Diseases of Women and Children (Sichuan University), Ministry of Education, Chengdu, Sichuan Province, China.”
